# Perception of AI Symptom Models in Oncology Nursing: Mixed Methods Evaluation Study

**DOI:** 10.2196/82283

**Published:** 2026-02-04

**Authors:** Bridget Nicholson, Elizabeth A Sloss, Aref Smiley, Joseph Finkelstein, Kathi Mooney

**Affiliations:** 1 College of Nursing, University of Utah, 10 2000 E, Salt Lake City, UT, United States; 2 College of Medicine, University of Arizona, 1401 University Boulevard, Tucson, AZ, United States; 3 College of Nursing, Huntsman Cancer Institute, University of Utah, Salt Lake City, UT, United States

**Keywords:** artificial intelligence, AI, oncology, nursing, symptom management, implementation

## Abstract

**Background:**

Patients undergoing cancer treatment experience a significant symptom burden. The standard process of symptom management includes patient reporting and clinical response following symptom escalation. Emerging predictive symptom models use artificial intelligence (AI) components of machine learning and deep learning to identify the risk of symptom deterioration, facilitating earlier intervention to prevent downstream effects. However, integrating predictive symptom models into clinical practice will require oncology nurses to adopt innovative approaches.

**Objective:**

This study aims to explore oncology nurses’ perceptions of the use of predictive symptom models in cancer care and the factors influencing the adoption of this symptom care innovation.

**Methods:**

The evaluation was guided by the Rogers Diffusion of Innovation Theory, which describes the process of how individuals adopt new technologies. The investigators developed an interview guide that asked oncology nurses to rate their perceptions of AI symptom models on a Likert scale. Participants were also asked to provide qualitative comments to support their ratings for each question, in order to better understand the key factors that would influence AI predictive model adoption. Investigators analyzed demographic data and Likert ratings with descriptive statistics. Qualitative analysis of participant comments included content analysis and inductive coding to identify themes. Nurses’ perception of factors that would influence the adoption of AI symptom models, based on the Rogers theory, included relative advantage, compatibility, complexity, trialability, and observability.

**Results:**

Responses of 15 oncology nurses with more than 1 year of experience in oncology were analyzed. There was high agreement among nurse participants that an AI model could improve symptom management for patients with cancer (n=10, 67%) and increase early intervention to prevent the escalation of symptoms (n=12, 86%). All participants (N=15) agreed that receiving symptom information would be helpful. Nearly three-quarters of participants (n=11, 73%) endorsed that the information would save time. Most (n=12, 80%) recommended that clinicians receive information about the predicted symptom deterioration of their patients. Among open-ended responses, key themes were consistent with factors identified in the Diffusion of Innovation theory including: (1) perceptions related to the AI model (compatibility or complexity), (2) nurses’ perception of patients' benefit (observability), (3) improved clinical processes (relative advantage or observability), (4) apprehension over model accuracy and impact (compatibility or trialability or observability), and (5) implementation or adoption (trialability or complexity or observability).

**Conclusions:**

Oncology nurses agree that predictive symptom models could help improve symptom management for patients undergoing cancer treatment. However, nurses noted that transparency in the factors included in the AI model was essential, that nurses should be involved in the development and testing of models, and that the observability of the benefit in symptom care would need to be evident for ultimate adoption.

## Introduction

Patients undergoing cancer treatment experience a wide range of symptoms that impact functional status, quality of life, and health care use [1-3]. Currently, symptom reporting is a reactive process based on patient reporting, followed by a response for poorly controlled symptoms. Oncology nurses have historically screened patients both at clinical visits and via phone triage when patients report increasing symptom burden [3]. Increasingly, but slowly, clinical workflows are implementing symptom monitoring with electronic patient-reported outcome (ePRO) systems, which enable patients to report symptoms electronically and allow oncology clinicians to respond accordingly [4]. However, the ePRO-based symptom management decreases care escalations, which is notable given that worsening of symptoms is a primary driver of health care use among patients with cancer [3]. However, oncology symptoms can change rapidly, and some, such as fever, require prompt evaluation and clinical action [5,6]. While responsive ePRO reporting systems have improved patient symptom burden, high levels of symptoms and health care use persist. ePRO symptom models remain reactive, with detection following a patient reporting a change and lacking the ability to anticipate symptom escalations.

One application of artificial intelligence (AI) is the use of computer-based models to analyze large quantities of data, in this case, symptom data. Predictive symptom models attempt to evaluate data and detect a change prior to patient symptom escalation. AI models are being tested using retrospective and prospective data [7]. AI models, paired with ePRO collection, are being developed to enable predictive and anticipatory warnings that may help categorize patients at an increased risk of symptom escalation [8]. AI models use patient-generated data to predict the likelihood of a specific outcome or a set of outcomes [8]. Models to diagnose both diseases and symptoms, as well as health care use, are being integrated into oncology use cases [7,9]. Predictive symptom models, which inform the identification of symptom patterns, show promise as a mechanism to enhance the accuracy of symptom detection before escalation [7,10]. AI-derived alerting models, using machine learning (ML) or deep learning methods, have the potential to predict emerging symptom escalations. These models seek to prioritize patients at increased risk for changes before symptom escalation. Detecting symptoms, such as an impending fever, before the patient experiences it can facilitate earlier intervention and better outcomes [11]. Alternative predictive approaches are necessary to detect dynamic symptom changes while reducing the burden of symptom reporting.

Transitioning to proactive care models requires a complete shift, both cognitively and operationally, for both patients and clinicians. Moving from a reactive reporting structure to a predictive symptom management model requires adoption by the oncology team. Notably, the implementation of this shift will require the engagement of oncology nurses, who will be the clinicians responsible for responding to AI-based alerts.

Few studies have examined nurses’ perceptions of implementing AI-based symptom models [12]. A recent study that assessed nurse perspectives on ML-based clinical decision support systems broadly found that previous experience with technology and nurse perceived engagement in the development process, among other factors, influenced perceived use of ML clinical decision support systems [13]. The use of AI in the clinical setting is expanding, and a key theme consistently identified by nurses, nurse informaticists, and nurse leaders regarding the development, implementation, and adoption of AI-based tools is the importance of engaging nurse end-users at the beginning of the development process [14-16]. Thus, the purpose of this evaluation was to examine nurses’ willingness to adopt AI-derived alert notifications about impending symptom escalations. In anticipation of implementing these AI-based symptom management systems, this exploration addresses an existing gap in the literature regarding oncology nurses’ perceptions, including usefulness and anticipated efficiency, of AI-derived symptom prediction models for cancer symptom management.

The Rogers Diffusion of Innovation Theory describes the process of how users decide to participate in the adoption of new technologies [17] and framed our work to nurses’ consideration to adopt AI. Using AI-based models in clinical practice will require a significant transition from current symptom evaluation processes, and oncology nurses, who are largely responsible for symptom triage, will need to adopt and use this innovation in care management workflows. Perception of the innovation, rather than the innovation itself, is key to adoption. The Diffusion of Innovation theory identifies 5 perceived attributes that influence adoption, including relative advantage, for this study whether the AI predictive models are perceived as improving current symptom monitoring and would benefit patients; compatibility—whether the AI predictive models are consistent with symptom treatment values, past experiences, and the needs of nurses providing symptom care; complexity—whether AI predictive models are seen as easy to understand and use; trialability—whether the AI predictive models can be piloted and tried out; and observability—whether the symptom management benefits of the AI predictive models can be seen by the nurses. According to the theory, adoption occurs at varying speeds based on individual characteristics and perceptions, such that a small percentage of the population will be innovators and early adopters, and others are more likely to adopt later after others have accepted the innovation. The focus of this study is on these factors and how they may influence nurses’ perceptions and decision-making about the adoption of AI predictive symptom models.

## Methods

### Design, Setting, and Participants

We conducted a mixed methods exploration of oncology nurses’ perspectives regarding the use of AI-based symptom predictive models to detect symptom changes in patients with cancer. The use of both structured questionnaire (eg, Likert-scale questions) and interview questions allowed for a more in-depth analysis of perspectives regarding the adoption of AI predictive symptom models and is well-suited for implementation research [18] Specifically, we conducted interviews with participants using both structured, Likert-scale–based questions and open-ended questions.

A convenience sample of registered nurses with at least 1 year of experience in oncology from across the United States was recruited to participate in this project. Participants were excluded if they lacked fluency in spoken or written English, lacked access to Zoom (Zoom Communications, Inc) web-conferencing technology or were unable to meet in person, or if they had less than 1 year of experience as a nurse in oncology. Recruitment methods included direct professional referrals, social media (such as LinkedIn and Facebook), and snowball sampling. Investigators contacted participants via email to schedule interviews. Interviews, the duration of which ranged from 20 to 30 minutes, were conducted in December 2024 and January 2025 via web teleconferencing platform (Zoom) and in-person by 2 investigators (BN and EAS). Interviews were not recorded or transcribed, though detailed notes were kept by the investigators who conducted the interviews and included capturing verbatim quotes from participants.

### Individual Interviews

The team developed the interview guide (Multimedia Appendix 1) to gather information on the acceptability of implementing AI predictive symptom monitoring and management. The interview guide was initially drafted by 2 investigators (BN and EAS) and feedback was obtained from other members of the team before being finalized (Multimedia Appendix 1). Before starting the interview, as outlined in the interview guide, the concept of an AI-based symptom model was presented to the participants. The description was broad in that it included general model types but emphasized the predictive capability of AI algorithms in the identification of symptom deterioration. In addition to demographic questions, the final interview guide consisted of 6 total Likert-scale questions, in which participants responded to statements about the hypothetical clinical usefulness and efficiency of a symptom prediction model, indicating their agreement or disagreement using the Likert scale (1=“Strongly Disagree“ to 5=“Strongly Agree”). Following each Likert-scale question, participants were asked to provide open-ended comments in response to the Likert-scale question that they had previously answered. Three additional open-ended questions were meant to elicit additional information, for example, “If you received a notification that your patient is at high risk for experiencing worsening symptoms in the next 24 hours, what would you do?”

Saturation was assessed on an ongoing basis. No new information was elicited, and subsequently, no new codes were identified over the final 5 interviews, indicating that we achieved content-level saturation.

### Data Analysis

#### Quantitative Analysis

Descriptive analyses, including means and SDs, were calculated using demographic data to describe the sample. Due to the small sample size, we rounded frequencies (percentages) to the whole number. Additionally, investigators evaluated the frequency of Likert ratings by participants through descriptive statistics. The Likert-scale ratings were on a 1 to 5 rating with responses initially coded based on a 1 to 5 rating (1=“Strongly disagree,” 2=“Disagree,” 3=“Neutral,” 4=“Agree,” and 5=“Strongly agree”). However, in further analyses, we combined ratings of 1 to 2 and 3 to 5 to create categorical ratings of “Disagree,” “Neutral,” and “Agree.” Methodologically, this approach is used to improve interpretability in smaller sample sizes, which have limited responses in multiple categories [19]. Our quantitative analysis of Likert-scale responses ultimately provided a clearer picture of the reportable trends within the sample.

#### Qualitative Analysis

For qualitative analysis, the team members (BN and EAS) used open coding and initially coded qualitative responses independently. After resolving disagreements and reaching consensus on codes, the investigators recoded each qualitative interview. Data were then analyzed using thematic analysis which involved several steps: data familiarization, keyword selection, identification of initial themes, and comparison of the investigators’ initial themes.

#### Triangulation

In keeping with a mixed methods approach, the investigators synthesized quantitative and qualitative data and identified findings that converged, complemented, or diverged across data modalities [20,21]. Quantitative data from Likert-scale responses were triangulated concurrently with qualitative, open-ended responses to the questions and/or follow-up prompts. Finally, the investigators compared the codes for the factors within the Diffusion of Innovation Model. Participant quotes were used to represent themes. We used the GRAMMS (Good Reporting of a Mixed Methods Study) guidelines to aid clarity of reporting (Checklist 1) [22].

### Ethical Considerations

The University of Utah Institutional Review Board reviewed the project protocol and deemed it a quality improvement project and not human participants research (00166873). Each participant was informed of the purpose of the project, including that participation was and could be discontinued at any time for any reason. Verbal consent was obtained prior to proceeding with the interview. No compensation was provided to the participants. In accordance with the rigor of human participants research, the study team followed procedures to protect the participants’ privacy and confidentiality, including deidentifying participant data, not sharing data outside of the study team, and storing data securely on password-encrypted computers.

## Results

### User Statistics

Sample characteristics are summarized in Table 1. The sample included 15 nurses who self-identified as working in oncology for more than 1 year. Participants were all female (15/15, 100%) with a mean age of 44.6 (SD 11.44) years. The cohort consisted of an experienced group of nurses, with an average of 18.33 (SD 9.82) years of nursing experience. Most of this experience (mean 14.10, SD 9.92 y) was in oncology. Participants reported working in diverse oncology settings, including inpatient oncology and outpatient infusion, as well as in roles related to quality improvement and patient navigation (Table 1). Furthermore, the sample was highly educated, with 8 out of 15 (53%) having completed a master’s degree (Table 1).

**Table 1.** Participant demographics (N=15).

Characteristics	Participants
Age (y), mean (SD)	44.6 (11.44)
Years of experience, mean (SD)	18.33 (9.82)
Years of experience in oncology	14.10 (9.92)
Highest level of education, n (%)	
Diploma	1 (7)
Bachelor’s degree	4 (27)
Master’s degree	8 (53)
Doctoral	2 (13)
Practice environment, n (%)	
Inpatient oncology	4 (26)
Outpatient oncology	8 (53)
Quality	1 (7)
Navigation	2 (13)

### Quantitative Evaluation

Results are presented in categorical (agree or disagree) percentages for the 6 Likert-scale questions (Table 2). All nurse participants overwhelmingly agreed that receiving the symptom information would be helpful, signaling compatibility with existing values. Furthermore, 12 out of 15 (86%) nurses believed that an AI model would enable early intervention to prevent the escalation of symptoms, aligning with this view. Most nurses (12/15, 86%) also thought that an AI model would allow the relative advantage of early intervention to prevent the escalation of symptoms. A smaller majority, or 10 out of 15 (67%) nurses, agreed that an AI model could improve symptom management for patients with cancer. The remaining one-third or 5 out of 15 (33%) participants were neutral about whether the symptom prediction model could help improve symptom management related to the disease. There was similar agreement on the expectation that a symptom prediction model would enhance a patient’s quality of life, with 10 out of 15 (67%) nurses agreeing. From an efficiency perspective, 11 out of 15 (73%) nurses felt that the information may save time. Despite nurses obtaining significant volumes of clinical information during a clinical day, 12 out of 15 (80%) nurses recommended that clinicians receive information about the predicted deterioration of their patients.

**Table 2.** Perceptions of artificial intelligence (AI) predictive ratings’ value in oncology symptom management (N=15).

Topic	Disagree, n (%)	Neutral, n (%)	Agree, n (%)
Knowing that a patient is at risk of symptom deterioration earlier is helpful information for me to have as an oncology clinician	0 (0)	0 (0)	15 (100)
I expect that information from an AI model would allow me to intervene earlier, preventing an escalation of patient symptoms.	0 (0)	2 (13)	12 (86)
I would recommend oncology clinicians receive information about predicted deterioration from an AI algorithm when caring for patients with cancer.	1 (7)	2 (13)	12 (80)
Having this information might save me time and/or help improve my efficiency in helping my patients reduce their symptom burden	1 (7)	3 (20)	11 (73)
I expect information from an AI model would add to reducing symptom burden and improve my patients’ quality of life.	2 (13)	3 (20)	10 (67)
I expect information from an AI model would help me better manage symptoms related to cancer treatment or disease.	0 (0)	5 (33)	10 (67)

Only 1 out of 15 (7%) respondents indicated that they believed AI-based symptom models would not improve efficiency or would not recommend that oncology clinicians receive information regarding patient deterioration from an AI symptom model. A small number, 2 out of 15 (13%) nurses indicated that AI-based symptom models would not reduce symptom burden or improve quality of life. These concerns reflect fears of complexity, given the complete shift in operational paradigm. A higher percentage of respondents, ranging from 13% to 33%, were neutral in their responses, indicating that they were still considering the information on the innovation.

### Qualitative Evaluation

#### Themes

**Figure 1.**
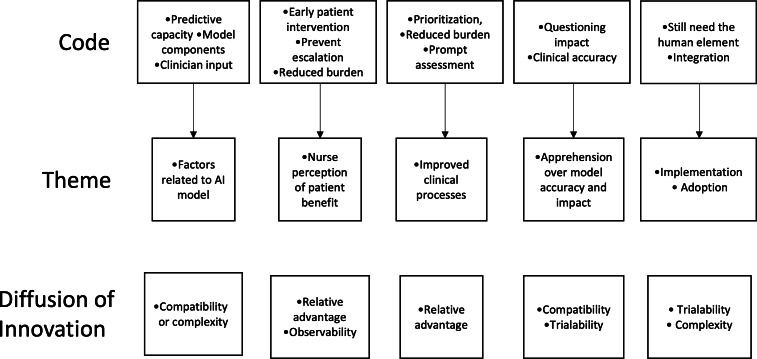
Codes, themes, and diffusion of innovation category. AI: artificial intelligence.

Participants’ comments further explained their perceptions about the development of symptom prediction models, including: (1) factors related to the AI model (compatibility or complexity), (2) nurse perception of patient benefit (relative advantage or observability), (3) improved clinical processes (relative advantage), (4) apprehension over model accuracy and impact (compatibility or trialability), and (5) implementation or adoption (trialability or complexity). Table 3 displays the identified themes, perceptions influencing adoption, and select quotes. Figure 1 highlights the development of themes from codes and the attributes of the Diffusion of Innovation Theory.

**Table 3.** Themes, codes, participant number, and quotes.

Themes	Perception attributes	Codes	Exemplar quote(s)
Factors related to the AI^a^ model	Compatibility Complexity	Predictive capacity Model components Clinician input	*I think it might help if the algorithm says the patient is likely to develop a fever. What would I do with that information if there were no infectious symptoms? It might be helpful if managing a lot of patients helps someone to rise to the top for check-ins.* [Participant 11] *Depends on where they get their data from. We already know when chemotherapy induced nausea will occur... so if AI is using appropriate standardized resources, I would be fine with that.* [Participant 8]
Nurse perception of patient benefit	Relative advantage	Early patient intervention Prevent escalation Reduced burden	*...a lot of time patients wait until symptoms are worse to call. We can intervene sooner.* [Participant 14] *Oncology patients can deteriorate quickly, this could help get symptoms before out of control to avoid ER/ hospital visit.* [Participant 1] *If a [model] did exist I would highly recommend [it] to prevent or mitigate life events to help prevent life or death.* [Participant 6]
Improved clinical processes	Relative advantage	Prioritization Reduced burden Prompt assessment	*Prioritization should occur digitally, rather than me doing it, adding more to my work burden.* [*Participant 12*] *Delays in care, due [to patient] burden of calling in. The RN reaching out directly could increase patient satisfaction and response.* [Participant 12]
Apprehension over model accuracy and impact	Compatibility Trialability Observability	Questioning impact Clinical accuracy	*Based on clinical practice, you can usually pinpoint those patients anyway.* [Participant 12] *I don’t really know if I would fully trust every time until it proves itself.* [Participant 3]
Implementation or adoption	Complexity Trialability Observability	Still need the human element Integration Workflow Communication of model output (eg, notifications, text) Ease of use Alert fatigue	*AI can’t supersede one-on-one contact.* [Participant 10] *[I] would want notifications if they are interruptions… I would want [them] to be relevant.* [Participant 12]

a AI: artificial intelligence.

#### Factors in the AI Model

Themes focused heavily on nurses’ ability to understand the factors within the model and test it to assess its predictive capacity, components, and provide input into its development. Predictive capacity refers to the model’s ability to make accurate assessments of future behavior. For example, participants emphasized the importance of the model being accurate and relevant to the patient’s clinical presentation, reflecting the need for compatibility with existing systems. For example, one participant stated:

*[I would want to know] … what led to notification, reason behind alert… what was their trend before… algorithm that shows patients who exhibit X also show Y, in the context of what’s going on with the specific patient*.

[Participant 15]

Participants also commented on specific factors necessary as model components, such as temperature and respiration. Nurses strongly emphasized the importance of involving oncology clinicians in the development of the AI model, highlighting their need to understand its compatibility with current systems.

#### Patient Benefit

Another theme identified was the benefits to patients, which included codes for early patient intervention, prevention of escalation, and reduced patient burden, all of which are compatible with current systems. Early patient intervention, as noted by many participants, was identified as a benefit of AI-based symptom management and is defined as having contact with the patient in a manner that occurs earlier than standard care as a relative advantage. For example, participants noted that a model could allow them “to intervene earlier before symptoms progress into dangerous situations” (Participant 2) or “prevent hospitalization and improve quality of life by managing symptoms at home” (Participant 8). Early intervention is the mechanism by which ePRO alerting systems have effectively decreased escalations of care from a current setting to a higher level of care, such as an emergency room. Other participants disagreed that patients would benefit more than they already do, with one participant stating:

*I don’t think the AI model will provide much additional information...Nurses already watch for specific symptoms.*

[Participant 13]

Nurse participants also focused heavily on reducing the burden of cancer care delivery on patients. They highlighted the fact that the combination of early intervention, for example, early symptom detection, can prevent later-stage symptoms and care escalations, thereby improving the experience of cancer care, which aligns with the goals of current systems, but may also represent perceived advantages over the current system.

#### Enhanced Clinical Processes

Participants could envision that an AI-based symptom model may enhance the process of clinical care by improving prioritization and response times, thereby facilitating the prompt assessment of clinically significant changes in a manner superior to current systems. Additionally, participants felt that the process of care could decrease clinical burden, for example, stating “being able to streamline information would be helpful” (Participant 7). Nurses also reported wanting to reduce the patient’s need for reporting and the burden of care escalations to clinicians. However, some participants also expressed concerns that the model could increase clinical burden and highlighted concerns about complexity, noting:

*The nurse will have to contact the patient. Just because they have an alert doesn’t mean they will have the symptoms.*

[Participant 10]

#### Model Accuracy

Participants also reported it would be imperative to test the model to verify its accuracy, noting that nurses would be more likely to use a model they could participate in testing. Participants cautioned that patient engagement may influence the clinical accuracy of the tool. Many nurses have progressed beyond the initial knowledge stage and are now considering not only whether, but also how, systems should adopt AI symptom models. Nurses have experience in integrating new technologies into clinical practice; as such, they understand the importance of accepting innovation to facilitate its diffusion and optimal use. Nurse participants also reported some apprehension about the use of AI models. Specifically, participants voiced concerns about the effectiveness and clinical use. Participants noted that training the model with the correct inputs would be crucial in confirming the model’s accuracy.

#### Implementation Processes

Nurses’ comments emphasized that decision-making also depends on the practical implementation of AI-based models. Evaluation and trialability of escalation alerts would be necessary for both initial and long-term adoption. Participants reported that AI-based predictive alerts for symptom management will not replace human nurse assessment and response. Participants also noted that the integration into the workflow needs to be seamless. There were many comments related to the importance of ensuring that communication of model output to nurses and other clinical staff does not increase the time burden, though many thought it would. For example, one participant noted, “I don’t know it will save time, [it] may add time, but that is the sacrifice to catch something early” (Participant 12). Furthermore, most participants expressed a firm belief that a model could be easy to use and would not contribute to alert fatigue.

## Discussion

### Principal Findings

The majority of our sample of nurses agree with statements that support the use of AI-based symptom models, reflecting nurses’ belief that these models may represent a relative advantage to current practice. The themes that nurse participants identified as essential to the adoption of AI symptom models aligned with the Diffusion of Innovation Theory. Nurses have recognized that the compatibility of AI-based symptom models holds promise for predicting, detecting, and enabling a response to changes in patient symptoms. Nurses’ strong agreement to receive symptom information via new models revealed an overall favorable view of this type of model and alignment with existing values. These models align with nurses’ strong commitment to providing patients with the best possible care, and by fostering the potential for AI-based symptom management models to improve patient care. Specific benefits identified by participants include improving clinician response by increasing the information clinicians receive and reducing patient burden through the elimination of unnecessary reporting or care escalations. This type of agreement indicates that nurses have progressed beyond the knowledge stage in the innovation process, toward identifying the necessary information to adopt the use of models. Overall, oncology nurses have positive views regarding the perceived advantages for patients and the compatibility with current care. This study demonstrates that many nurses have positive perceptions of the advantages and usability of AI-based symptom models and are now considering the implementation and use beyond the potential value.

Despite support for adoption, nurses urged caution in the development and implementation of these models. In particular, nurses emphasized the importance of involving end-users in the development, pilot testing, and implementation of these models, as this will help determine their value and appropriate integration into clinical workflow, thereby facilitating their adoption. Nurses have experience in integrating new technologies into clinical practice; as such, they understand the importance of accepting innovation to facilitate its diffusion and optimal use. This aligns with a framework developed for designing and implementing AI models from a systematic review, which recommends the inclusion of health care providers in development and implementation [8].

Nurse participants recognized the importance of trialability through accurately training and testing models, as well as ensuring that the data sources used are adequate to positively impact patient outcomes. Nurses strongly felt that model development requires the careful selection of clinically appropriate inputs, such as the inclusion of temperature and laboratory values, to support clinically accurate results. Confirming models that are appropriate for the input data and the desired outcomes is necessary for accuracy. As frontline users, nurses who currently assess patient symptoms should be included in model factor selection and testing. Often, these models are developed in collaboration with other clinical providers, and yet nurses will be the ones to receive the alerts and need to triage them. Trialability and observability for nurses, not just physicians, are keys to adoption. These themes are consistent with the recommendations for transparency in the development of AI-based clinical models, ensuring that both clinicians and patients understand and agree on the inputs to the model [23]. Creating transparent and explainable models is a step toward combating the perpetuation of healthcare bias in AI models and will facilitate long-term adoption [24,25].

While participants identified the need to understand model inputs and testing, they also reported a need to see the model’s impact on outcome to feel confident in making clinical decisions based on the model, again underscoring the importance of observability. Model outcome achievement depends on the implementation of models as designed. For this to occur, there must be transparency and trialability of both inputs and clinical outcomes. For example, our early work developing a predictive model demonstrated the ability to predict symptom escalation more accurately in short intervals than at longer intervals [10]. Transparency will enable clinical teams to implement models for the purpose they were developed, thereby supporting accuracy. Efforts to transform and train models for additional uses will need to follow proper rigor to ensure the models are adapted and updated effectively. Transparency and inclusion in development will enable oncology nurses to effectively use AI-based models.

Experienced oncology nurses in our sample reported both a strong interest in using and some reluctance to immediately trust AI-based symptom models. While involving nurses in the development and implementation will facilitate trust, oncology nurses may lack the education and training to understand how these models work. An extensive national survey of nurses revealed that only 30% of nurses are aware of how AI is used in nursing practice [26]. Although information regarding the use and daily applications has increased in the last several years, this highlights the need to provide AI education to both students and to disseminate it to nurses at the point of care delivery. Future work should specifically evaluate the education needs of oncology nurses regarding AI-based symptom models.

With many clinical symptom escalation models still in development, gaining a clear understanding of nurse perceptions regarding the use, decision to adopt, and maintenance of these models is essential. Our examination revealed that oncology nurses share similar concerns to those documented in the literature regarding the use of clinical predictive models, including alert fatigue and increased time burden, which represent a source of complexity [27]. Additional barriers to adoption of AI technology in healthcare include ethics, technological considerations such as data access and infrastructure, and liability and regulatory issues [28]. However, as evidenced in our results, nurses also hold favorable perceptions that these models have advantages and align with current treatment values, prioritizing the reduction of cancer symptom burden. Implementation strategies that could be used to overcome adoption barriers include, but are not limited to, identifying implementation champions as well as ensuring adequate interpretability of the model [29,30]. AI-based symptom models have the potential to improve patient outcomes and enhance clinical processes when implemented thoughtfully into the clinical workflow. As the end users of AI-based symptom management models, nurses should be involved as content experts, beginning with model development and continuing through the design, integration, and evaluation of the model into workflows, to maximize both short-term implementation and long-term adoption. However, additional research is needed to identify which implementation strategies are effective at promoting the adoption and sustained use of AI-based symptom management models.

### Limitations

We sought to elicit oncology nurses’ initial thoughts on AI-based symptom prediction models. We should continue to inductively evaluate nurses’ adoption of AI. Our exploration was limited by a small sample size and a homogenous population that was skewed by age (older) and education levels (high) that impacts the generalizability of our findings. This may be attributable to our convenience sampling approach and the fact that participants recommended other individuals who were recruited to participate, possibly introducing selection bias. Both the sample skew and homogeneity may have influenced the overall positive perceptions of an AI model for use in symptom management. Additionally, the use of unrecorded and note-based qualitative data analysis may have limited our ability to accurately assess content-level saturation; however, we believe that the detailed notes taken by interviewers permitted accurate assessment of content-level saturation. Finally, while we used the Rogers Diffusion Theory of Innovation to improve the descriptive analysis of the qualitative themes, it may limit our understanding of the responses and future work. Further work should survey a larger sample of nurses to understand oncology nurses’ perceptions of AI symptom models and consider the impact of education levels on their views regarding AI. Additionally, future work should highlight the gaps in nurses’ understanding of the application of AI in clinical care. The inclusion of end users in the design and testing of AI-based models facilitates adoption, and additional work should concentrate on and focus on implementation processes, which include user-centered design testing of best practices, such as alerting, alert visualization, and responses to care.

### Conclusions

Overall, nurses showed a positive attitude toward the adoption of AI-based symptom models, particularly highlighting the perceived advantages of such models and their compatibility with nurses’ goals of enhancing the patient experience. Proper use of AI symptom prediction models creates the opportunity to decrease the burden of patient reporting of cancer symptoms, improve clinician responsiveness, and enable prompt intervention to reduce unnecessary care and escalations. To facilitate the seamless integration of AI-based symptom models, thoughtful inclusive design strategies must include end users to test and modify transparent clinical models for long-term adoption.

## Supplementary material

10.2196/82283 Multimedia Appendix 1 Symptom Care at Home (SCH) artificial intelligence (AI) model interview script.

10.2196/82283 Checklist 1 GRAMMS Checklist.

## Abbreviations

AI: artificial intelligence
ePRO: electronic patient-reported outcome
GRAMMS: Good Reporting of a Mixed Methods Study
ML: machine learning
