# Supplementary material for: Perception of AI Symptom Models in Oncology Nursing: Mixed Methods Evaluation Study
Source: JMIR Nurs. 2026 Feb 4;9:e82283. doi: 10.2196/82283 (PMC12871576; doi:10.2196/82283)
Supplement: Multimedia Appendix 1 [file nursing-v9-e82283-s001.docx]

**Interview Script**

**Interviewer:** Hello and thank you for participating in this interview for our project. Before we get started, I would like to provide some context on what we are interested in learning from you about. As you may know, electronic patient reported outcome systems (ePROs) exist that allow patients to report symptoms related to cancer treatment and disease burden. One of these systems is called Symptom Care at Home (or SCH). The SCH system asks patients report symptoms daily. Currently, we are exploring using patient-reported symptom data from thousands of patients to develop an artificial intelligence model, or algorithm, that can predict when patients are likely to experience a symptom escalation based on symptom patterns. The idea is that this AI algorithm could anticipate or detect deterioration before a physical sign or symptom is present in the patient and could be used to inform early intervention to prevent symptom escalation.

The goal of this project is to understand if this type of information would be useful to you as a practicing clinician. For example, if you were notified that an AI algorithm detected that patient is at risk for an adverse event, do you think this could improve your clinical care? We are interested in your thoughts on the application of these methods to clinical care, and if it could help you help save you time or delivery more efficient care.

Do you have any questions before we move forward with the interview?

*[Pause]Interviewer notes: For I and II, if people give a low score I would follow up and ask them why they think it would not be helpful and if they proved a highly positive score, I would ask them how it would be helpful*.

**Part 1: Usefulness:**

*Usefulness perceptions: Measure expected outcomes of sharing this information with oncology clinicians.*

**Interview:** I would like to get your feedback on how useful you think an AI algorithm as I’ve described is when taking care of oncology patients.

On a scale of 1 to 5, where 1 is strongly disagree and 5 is strongly agree, please rate your agreement of the following statements:

“I expect that information from an AI model would:

1. Help me to better manage symptoms related to cancer treatment and disease. (1-5)
2. Allow me to intervene earlier, preventing an escalation of patient symptoms.
3. Prevent unnecessary escalations of care, such as unplanned hospitalization
4. Add to reducing symptom burden and improve my patient’s quality of life

Is there any feedback that you would like to share based on the statements thus far?

**Probing question:** If you received a notification that your patient is at high risk for experiencing worsening symptoms in the next 24 hours, what would you do?

What would be important to you in receiving a notification that was based on a AI model?

**Part 2: Acceptability:**

*Measure perceived acceptability of the content in the tool to support a prognosis discussion.*

**Interviewer:** Please rate your agreement on a scale of 1 to 5 with 1-strongly disagree to 5-strongly agree.

1. Knowing that a patient is at risk of symptom deterioration earlier is **helpful** information for me to have as an oncology clinician.
2. Having this information might **save me time** and/or **help improve my efficiency in helping my patients to reduce their symptom burden**.
3. **I would recommend** that oncology clinicians receive information about predicted deterioration from an AI algorithm when caring for cancer patients.

Is there any additional feedback that you would like to share?

**Part 3: Expectations**

1. **What would you want or expect in order to feel comfortable with utilizing notifications based on an AI symptom model?**

Part 4: Closing

Is there anything else that you would like us to know or to share with us related to this project?

**Interviewer:** Thank you for participating in this interview.
