# Supplementary material for: Perception of AI Symptom Models in Oncology Nursing: Mixed Methods Evaluation Study
Source: JMIR Nurs. 2026 Feb 4;9:e82283. doi: 10.2196/82283 (PMC12871576; doi:10.2196/82283)
Supplement: Checklist 1 [file nursing-v9-e82283-s002.docx]

**Good Reporting of A Mixed Methods Study (GRAMMS) Checklist**

| **Guideline** | **Section: Page** |
| --- | --- |
| Justification to use a mixed methods approach to the research  question | Methods: Design, Setting, and Participants; page 4 |
| Articulation of the design in terms of purpose, priority, and sequence of methods | Methods: Design, Setting, and Participants, Individual Interviews, and Data Analysis; pages 4-5 |
| Describe each method in terms of sampling, data collection and analysis | Methods: Design, Setting, and Participants; page 4 |
| Delineate where and how integration occurs and who has participated in it | Methods: Data Analysis; page 5 |
| Describe any limitation of one method associated with the presence of another | Discussion: Limitations; page 13 |
| Describe insights gained from mixing or integrating methods | Discussion; page 12 |

O'Cathain A, Murphy E, Nicholl J. The quality of mixed methods studies in health services research. J Health Serv Res Policy. 2008;13: 92-98.
